# Effort Expenditure Reduces Prosocial Decision‐Making: Computational Principles and Neural Mechanisms

**DOI:** 10.1002/hbm.70290

**Published:** 2025-07-19

**Authors:** Yaxin Zhang, Jiarui Dong, Ningxuan Chen, Ping Wei

**Affiliations:** ^1^ Beijing Key Laboratory of Learning and Cognition and School of Psychology Capital Normal University Beijing China; ^2^ Faculty of Health and Wellness City University of Macau Macau SAR China

**Keywords:** dictator game, effort, monetary cost, prosocial behavior

## Abstract

Charitable giving is a costly prosocial act in which individuals donate money or other resources to benefit others. Although the relationship between effort and prosocial behavior has been explored, how effort expenditure affects subsequent prosocial decisions and the underlying neurocognitive processes remains poorly understood. We conducted two experiments to address this, using cognitive modeling of behavioral responses in Experiment 1 and electrophysiological recordings in Experiment 2. In both experiments, participants received cues indicating the effort type required (effort vs. no‐effort) before completing a task involving either physical effort or rest. They earned monetary rewards based on performance or unconditionally and then decided whether to accept donation offers at low, medium, or high costs. Behavioral results in both experiments revealed that participants were more likely to reject donation offers after exerting effort, particularly for medium‐ and high‐cost offers. Analysis using a hierarchical drift diffusion model revealed that participants accumulated information more rapidly and required less evidence for decision‐making in the effort condition compared to the no‐effort condition. Electrophysiological results revealed that effort expenditure heightened reward‐sensitive neural responses upon receiving monetary feedback, as reflected by increased reward positivity, fb‐P3, and fb‐delta power. Moreover, higher amplitudes of reward positivity and fb‐P3 in response to effort‐earned feedback were associated with less generous prosocial donations. These findings demonstrate that effort expenditure amplifies reward sensitivity, expedites the accumulation of self‐interest, simplifies the decision‐making process, and ultimately strengthens proself choices during decision‐making.

## Graphical abstract

Using cognitive modeling and electrophysiological recordings, we discovered that participants were more likely to reject high‐cost donations made with money earned through effort. We observed stronger neural responses in effort conditions compared to no‐effort conditions, and reward‐sensitive neural responses correlated with less generous donations after effort expenditure.

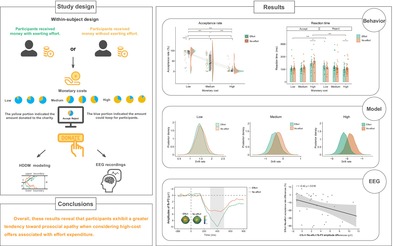

## 1 Introduction

Prosocial behavior refers to the positive actions that individuals consciously and voluntarily engage in to benefit other individuals, groups, or society as a whole (Bierhoff 2002; Penner et al. 2005). These processes often involve evaluating trade‐offs between personal costs, such as time, money, and/or effort, and the benefits to others (Apps et al. 2016; Contreras‐Huerta 2023; Contreras‐Huerta et al. 2020; Ferguson et al. 2019). Charitable giving exemplifies such costly prosocial behavior in real‐world contexts, wherein individuals donate money to organizations despite personal financial cost.

In laboratory settings, the dictator game (DG) is a commonly used paradigm to study charitable giving (Carlson et al. 2016; Cartwright and Thompson 2023; Filkowski et al. 2016; Forsythe et al. 1994; Li et al. 2021). In this paradigm, the “dictator” receives a sum of money prior to the task and is instructed to divide this fixed amount between themselves and a “recipient,” who must unconditionally accept the allocation. Due to the constant‐sum nature of the task, the dictator incurs a monetary cost to act prosocially toward the recipient. This paradigm has been employed in numerous studies and has provided critical insights into the factors that influence individuals' willingness to donate to charity. On one hand, the willingness to donate to charity is influenced by personal traits, such as affective empathy (e.g., Edele et al. 2013; Schaefer et al. 2021) and gender (e.g., Eckel and Grossman 2008; Kamas and Preston 2015, 2021; Mesch et al. 2011). On the other hand, donation decisions are also influenced by context‐dependent factors, such as money priming (Li et al. 2021), induced emotional state (Pérez‐Dueñas et al. 2018), social norms (Eckel et al. 2023), awareness of need (Huang et al. 2021), and acute stress (Azulay et al. 2022; Forbes et al. 2024; see Nitschke et al. 2022 for a recent review).

However, traditional DG paradigms differ significantly from real‐life charitable giving in one crucial aspect: participants typically donate experimenter‐provided “windfall” funds rather than personally earned money (Carlson et al. 2016; Huang et al. 2021; Li et al. 2021; Pérez‐Dueñas et al. 2018; Schaefer et al. 2021). In contrast, real‐world donations involve giving away income earned through physical or cognitive effort, which is often perceived as costly and aversive (Kool and Botvinick 2018; Kurzban 2016; but see Inzlicht et al. 2018). Recent research has identified “prosocial apathy,” wherein individuals demonstrate greater reluctance to exert effort for others' benefit compared to their own (Contreras‐Huerta et al. 2022; Depow et al. 2022; Forbes et al. 2024; Lockwood et al. 2022; Lockwood, Ang, et al. 2017; Talbot et al. 2025). While these studies primarily examine effort expenditure during prosocial acts themselves, less attention has been given to how prior effort expended in earning money subsequently affects donation decisions.

Some researchers have begun incorporating real effort tasks into their experimental designs, requiring participants to earn money before making prosocial decisions (Carlsson et al. 2013; Hernandez Lallement et al. 2014; Li et al. 2019). For example, Hernandez Lallement and colleagues manipulated cognitive effort (high effort, low effort, or no effort) through arithmetic tasks of varying difficulty before charitable donations, finding that participants were less willing to donate monetary rewards earned through high effort compared to those obtained effortlessly (Hernandez Lallement et al. 2014, Supplementary Experiment; see also Li et al. 2019).

Despite these behavioral insights, mechanistic understanding remains limited, particularly regarding the temporal neurocognitive processes underlying prosocial decision‐making after effort expenditure. Key questions persist about how effort expenditure interacts with varying monetary costs and how neural responses during monetary feedback influence subsequent charitable giving. Prosocial decisions typically involve trade‐offs between self‐interest (proself) and altruistic (prosocial) motives (Falco et al. 2023; Numano et al. 2024), with the relative influence of these motives shaped by monetary costs. However, it remains unclear whether different levels of monetary costs (low, medium, high) systematically modulate this trade‐off and how effort expenditure interacts with these cost‐dependent shifts. Moreover, while recent studies suggest that effort can enhance subjective evaluations of subsequent monetary feedback (Bogdanov et al. 2022; Harmon‐Jones, Clarke, et al. 2020; Harmon‐Jones, Willoughby, et al. 2020; Ma et al. 2014; Yi et al. 2020; but see Bowyer et al. 2021; Wu and Zheng 2023), no investigations have explored how neural responses during the monetary feedback (either through effort expenditure or not) influence subsequent costly charitable giving decisions. Specifically, the neural mechanisms by which effort‐based monetary gains affect subsequent donating behavior remain poorly understood.

To address these gaps, we conducted two complementary experiments to characterize the neurocognitive processes underlying prosocial decisions, integrating cognitive modeling of behavioral responses (Experiment 1) and electrophysiological recordings (Experiment 2). Both experiments employed the modified Monetary Incentive Delay (MID) paradigm (Knutson et al. 2000) and the DG paradigm (Forsythe et al. 1994), differing only in stimulus presentation and response collection methods to accommodate the specific requirements of computational modeling and electrophysiological recordings, respectively. In both experiments, participants were first presented with either an effort or a no‐effort cue, followed by a task requiring physical effort or rest. Participants received monetary rewards based on their performance in the effort condition, while they received monetary rewards unconditionally in the no‐effort condition. Subsequently, participants received a donation offer (involving low, medium, or high cost), where the endowment was divided between themselves and a charity, and they chose to accept or reject the offer.

The presentation of donation offer and the required responses varied between the two experiments. In Experiment 1, participants were instructed to respond with either acceptance or rejection when the donation offer was presented. We employed the hierarchical drift diffusion model (HDDM) to capture the differences in the evidence accumulation process between two option boundaries (accept vs. reject), utilizing a hierarchical Bayesian approach to estimate model parameters (Ratcliff and McKoon 2008; Wiecki et al. 2013). In contrast, in Experiment 2, we divided the decision process into two consecutive phases: the donation presentation phase and the response phase. This modification disrupted the evidence accumulation from the moment the donation offer was presented to the actual behavioral responses, rendering it unsuitable for the aforementioned modeling used in Experiment 1. However, this modification allowed us to more precisely capture the electrophysiological responses elicited by the donation offer, free from the confounding influence of subsequent behavioral responses.

In both experiments, we predicted a lower acceptance rate for charitable donations in the effort condition compared to the no‐effort condition, and this difference would become larger as the monetary cost increased. For Experiment 1, the parameters of HDDM include the following: drift rate (*v*), which represents the speed of the evidence accumulation process; decision threshold (*a*), indicating the distance between the two decision boundaries or the amount of evidence required for decision‐making (e.g., accept vs. reject); and non‐decision time (*t*), which encompasses the time for stimulus encoding and motor execution. Additionally, the model allows for a bias (*z*) that affects the starting point of the drift process relative to the two decision boundaries (Ratcliff and McKoon 2008; Wiecki et al. 2013). We examined the patterns of information accumulation as participants made binary donation choices while simultaneously assessing the effects of effort expenditure and monetary cost. We predicted that participants would have a higher drift rate (*v*) for rejecting high‐cost donation offers in the effort condition compared to the no‐effort condition. Additionally, we expected that effort would influence the threshold/boundary (*a*), leading to less evidence required to reach the decision boundary when participants exerted effort compared to when they did not. Given that participants would not be able to predict whether a low‐, medium‐, or high‐cost offer would be presented across trials, we hypothesized that monetary cost would not affect the bias (*z*). As a control measure, we also anticipated that the non‐decision time (*t*) would remain consistent across the models.

For Experiment 2, we utilized both event‐related potentials (ERPs) and event‐related spectral perturbations (ERSPs) analyses to investigate how physical effort and monetary cost influence the neural dynamics underlying charitable donation decision‐making processes. Despite being less widely adopted in charitable giving studies, the electrophysiological technique provides valuable insights into the dynamics of the neural processes involved in social decision‐making (Amodio et al. 2014; Helfrich and Knight 2019; Ibanez et al. 2012). We predicted the following results underlying effort anticipation, reward evaluation, and cost–benefit evaluation processes. In the effort anticipation phase, we hypothesized that signals reflecting the initial evaluation of anticipatory information (i.e., cue‐P2, cue‐delta) and/or signals associated with task preparation (i.e., contingent negative variation: CNV, cue‐alpha) would be more pronounced in the effort condition than in the no‐effort condition. In the reward evaluation phase, we expected that effort expenditure would elicit stronger reward‐sensitive neural responses (i.e., increased reward positivity: RewP, fb‐P3, fb‐delta) compared to the no‐effort condition. Finally, in the cost–benefit evaluation phase, we anticipated that after exerting effort, participants would allocate more attentional and cognitive resources to assess personal costs and self‐interests (i.e., increased P3 amplitude and delta band power), particularly for donation offers with medium personal costs. The medium‐cost condition may present the greatest difficulty for participants in making a decision compared to the high‐cost condition, which may easily lead to a rejection decision, and compared to the low‐cost conditions, which may easily lead to an acceptance decision.

## 2 Methods

### 2.1 Participants

We calculated the required sample size based on a medium effect size (Cohen's *f* = 0.25) and a power (1−*β*) = 0.95 for the *F*‐tests. The power analysis, conducted using G*Power (www.gpower.hlu.de/en.html), indicated that a minimum sample size of 28 participants was necessary to achieve 0.95 power with an *α* of 0.05.

A total of 30 participants took part in Experiment 1, while another 34 participants were involved in Experiment 2. The data from two participants in Experiment 1 were excluded due to a lackadaisical experimental attitude: one participant misunderstood the instructions, leading to incorrect color matching with decisions, and another participant had a high proportion of trials with donation decision times exceeding 4000 ms (more than 7%). In Experiment 2, data from one participant were discarded due to excessive eye blinks or muscle artifacts (see the EEG Analysis section for more details).

In Experiment 1, the remaining participants included 19 females and 9 males, aged between 19 and 28 years. In Experiment 2, there were 22 females and 11 males, aged between 18 and 29 years. All participants in both experiments were right‐handed, had normal or corrected‐to‐normal vision, and had no known cognitive or neurological disorders. The study was approved by the Ethics Committee of the School of Psychology at Capital Normal University, and all participants provided informed consent prior to the experiments, in accordance with the Declaration of Helsinki.

### 2.2 Design and Materials

A 2 × 3 within‐participant factorial design was employed in both experiments, with effort type (effort, no‐effort) as the first factor and monetary cost level (low, medium, and high) as the second factor. Both experiments were conducted in sound‐attenuated and standardized laboratory settings. Participants were seated comfortably at a distance of 65 cm from the screen in a quiet room, with light adjusted to fit. Stimulus presentations and response collection were performed using the PsychoPy 3 software package (http://www.psychopy.org).

### 2.3 Experimental Procedure

The calibration task preceding the formal experiment was adapted from Lockwood, Hamonet, et al. (2017), and the physical effort task was modified from the Effort Expenditure for Rewards Task (Treadway et al. 2009). Specifically, in the calibration phase for both experiments, participants were instructed to press the “A” key on the keyboard as many times as possible using the index finger of their non‐dominant hand for a duration of 4000 ms, performing this task three times. The average number of key presses across these attempts served as each participant's baseline for the Prosocial Effort task (see below). Subsequently, participants were required to select a public welfare project in Alipay philanthropy, which served as the charitable target in the Prosocial Effort task (see below).

**FIGURE 1:**
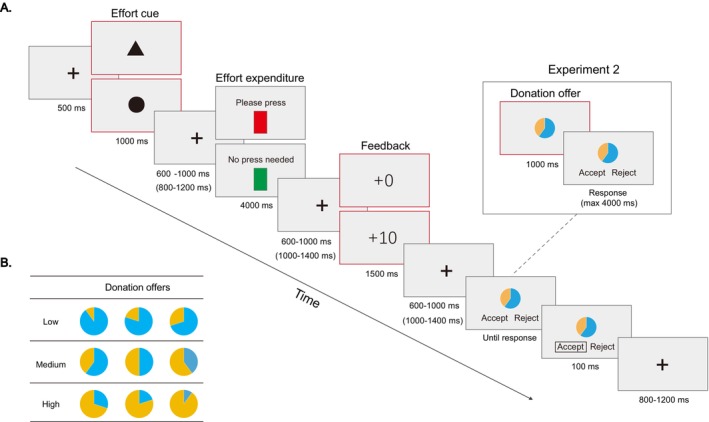
Trial structure and stimuli example. (A) The trial outline for the Prosocial Effort task. The experimental procedures for Experiments 1 and 2 were largely similar, with only minor differences. In Experiment 2, the interstimulus interval with fixation presented was adjusted to 800–1200 ms and 1000–1400 ms (as indicated in parentheses). Additionally, the donation frame was divided into two screens: The first screen displayed the donation offer for 1000 ms, followed by a response frame in which participants could make their choice to accept or reject the offer within 4000 ms. The red borders indicate the phases during which EEG activity was analyzed and reported in Experiment 2. (B) Examples of donation offers with low‐, medium‐, and high‐cost used in both experiments. In this example, the yellow portion indicated the amount donated to the charity, while the blue portion indicated the amount the participant could keep for themselves. The meanings of the yellow and blue portions were counterbalanced between participants.

An overview of the trial structure for the Prosocial Effort task is provided in Figure 1A. Each trial began with a fixation cross displayed at the center of a black screen for 500 ms. Following this, a cue (either a solid triangle or a solid circle) was presented for 1000 ms, indicating the type of effort required for the current trial (effort vs. no‐effort). The effort cue informed participants whether they needed to exert physical effort in the subsequent task.

After a variable interval of 600–1000 ms, participants were shown either a red rectangle with the instruction “Please press” (for the effort condition) or a green rectangle with the instruction “No press needed” (for the no‐effort condition), displayed for 4000 ms. In the effort condition, participants were instructed to press the “A” key with the index finger of their non‐dominant hand as many times as possible; in the no‐effort condition, participants were instructed not to press the key.

Next, after a jittered interval (600–1000 ms), during which a fixation cross was presented, visual feedback regarding their previous performance was displayed. A trial was deemed unsuccessful if the number of key presses did not exceed the baseline number from the calibration session, resulting in a feedback stimulus of “+0” displayed for 1500 ms before the next trial began. Conversely, if the number of key presses exceeded the baseline or if the participant was not required to press the key (no‐effort condition), feedback of “+10” would be shown for 1500 ms, indicating a monetary reward of 10 yuan for the participants.

Following the reward feedback of “+10,” an inter‐stimulus interval with a fixation cross was displayed for 600–1000 ms. Participants were then presented with donation offers categorized as low‐cost (1–3 yuan), medium‐cost (4–6 yuan), or high‐cost (7–9 yuan, Figure 1B). They were instructed to press the “K” key to accept the offer or the “L” key to reject it, using their right index or middle finger (the response buttons were counterbalanced across participants). Upon responding, the chosen option would be highlighted with a white border for 100 ms. Participants were informed that if the offer was accepted, the designated amount of money would be donated to the chosen charity, and the participant would retain the remaining amount; if the offer was rejected, the participant would keep the entire 10 yuan.

A new trial would then begin after an inter‐trial interval of 800–1200 ms, during which a fixation cross was displayed. Variable intervals were employed to prevent participants from developing time‐based expectations regarding the onset of subsequent stimuli. Both the color of the stimuli and the response buttons were counterbalanced across participants. To ensure that there were a sufficient number of trials with positive feedback in the effort condition, the baseline number of key presses per trial was controlled using a self‐adaptive procedure (Garcia‐Perez 1998, 2011). Specifically, the initial baseline number was the mean number of presses recorded during the calibration phase; then, for each trial in the effort condition, the baseline number would increase by 1 or decrease by 3 depending on whether the participant received positive or negative feedback, respectively, in the previous trial. This adaptive adjustment was designed to maintain the success rate at around 75% in the effort condition, thereby producing a comparable number of trials with positive feedback across effort and no‐effort conditions for subsequent analysis.

Accordingly, we set up a total of 315 trials in the formal experiment, including 135 no‐effort trials and 180 effort trials, divided into 15 blocks of 21 trials (comprising 9 no‐effort trials and 12 effort trials, with one third of trials corresponding to each monetary cost condition). On average, participants did not receive monetary reward feedback in the effort condition in about 45 trials due to the adaptive control of feedback stimuli. After these trials in the effort condition were excluded, the experimental series contained approximately 270 trials in total, with each of the six experimental conditions (2 effort types × 3 monetary cost levels) containing about 45 trials. Trials within each block were presented in pseudo‐random order. Prior to the formal task, participants completed 9 practice trials to familiarize themselves with the task and the corresponding button responses. To emphasize the significance of each trial, participants were informed that one trial would be randomly selected to determine their actual donation to a charity project of their choice. Thus, the outcome of the selected trial would directly influence both their donation and the amount they would retain for themselves. Consequently, the final reward for participants included a base pay (45 yuan in Experiment 1 and 115 yuan in Experiment 2), along with potential earnings from the selected trial, making each trial critical in determining their overall compensation and contribution to charity.

In Experiment 2, several adjustments were made to the timing of various intervals: the interval between the cue and the target was set to 800–1200 ms. Additionally, the intervals between the effort expenditure and the reward feedback, as well as between the reward feedback and the donation offer, were modified to 1000–1400 ms to accommodate the EEG analyses. Furthermore, to facilitate the analysis of EEG responses to the donation offer in Experiment 2, participants were first presented with the donation offer for 1000 ms, followed by a decision frame lasting 4000 ms, during which they could choose to accept or reject the offer (see Figure 1A). As mentioned in the Introduction, since the offer presentation frame and the decision frame were separated, the HDDM model could not be employed in Experiment 2. Aside from these timing adjustments, the overall experimental procedure for Experiment 2 remained consistent with that of Experiment 1.

### 2.4 Behavioral Analysis

For both experiments, we excluded missed trials during the donation stage, as well as trials with reaction times (RTs) that fell outside of 3 standard deviations (SDs) from the mean for each condition and participant. This resulted in the removal of 1.19% of total trials in Experiment 1 and 1.54% in Experiment 2. Since the acceptance rate data exhibited severe skewness and boundary effects. Specifically, the data points clustered around extreme values, with acceptance rates accumulated near 0% in the high‐cost conditions and near 100% in the low‐cost conditions. In accordance with best practices for describing non‐normally distributed and skewed data (Habibzadeh 2017), we reported the median and inter‐quartile range (IQR) instead of the mean and standard deviations for the acceptance rates. We utilized a General Linear Mixed‐effect Model (GLMM) to examine the acceptance rates. In this analysis, effort level (effort, no‐effort), monetary cost (low, medium, or high), and their interactions were specified as fixed effects. Participant ID was considered as a random intercept, and random slopes for the fixed effects were incorporated to capture individual variability. We used a Linear Mixed‐effect Model (LMM) to analyze RTs. This model included effort level, monetary cost, decision type, and their interactions as fixed effects, with participant ID and slopes for the fixed effects as random effects. These models were fitted using the maximum likelihood estimation (MLE).

### 2.5 Hierarchical Drift Diffusion Model

The HDDM was employed to further investigate the mechanisms underlying the prosocial decision‐making process. We used each participant's response (accept or reject) to fit the HDDM, focusing primarily on the threshold and drift rate parameters. To achieve this, we implemented three different models that allowed the parameters (*v* and *a*) to vary based on effort types (effort, no‐effort) or monetary costs (low, medium, high). For each model, we estimated 5000 samples from the posterior distribution using Markov Chain Monte Carlo (MCMC) methods (Spiegelhalter et al. 2002; Wiecki et al. 2013), with 2000 samples burned to stabilize the model. We then assessed model convergence using the Gelman‐Rubin statistic (Gelman and Rubin 1992). This convergence statistic was calculated five times per model, with values closer to 1 (but less than 1.2) indicating convergence (Spiegelhalter et al. 2002; Gelman et al. 2013). Finally, we selected the best model based on the lowest deviance information criterion (DIC) (Spiegelhalter et al. 2002). For the statistical analyses, we employed Bayesian hypothesis testing (with 95% credible intervals) to analyze the model parameters.

### 2.6 EEG Recordings and Preprocessing

In Experiment 2, EEG data were collected from 62 scalp sites using Ag/AgCl electrodes embedded in an elastic cap, with electrode locations based on the extended International 10–20 system (Neuro‐Scan; Compumedics, El Paso, TX). During recording, the electrodes were referenced to the left mastoid and later re‐referenced to the average of the right and left mastoid potentials offline. Additionally, two channels were used to record the electrooculogram (EOG) for horizontal eye movements (placed on the outer canthus of each eye) and vertical movements (placed above and below the left eye) to monitor blinks and other eye movements. Impedance was maintained below 5 kΩ, and the data were sampled at a rate of 500 Hz.

Offline EEG data preprocessing was performed using the EEGLAB toolbox (http://sccn.ucsd.edu/eeglab) in MATLAB. The EEG data were re‐referenced to the average of the left and right mastoid electrodes, and the signals were filtered with a band‐pass of 0.1–40 Hz. Continuous data were visually inspected to manually remove time epochs containing high‐amplitude, high‐frequency muscle noise and other irregular artifacts. Bad channels were interpolated using a spherical interpolation method, and independent component analysis (ICA) was applied to correct for eye‐blink and movement artifacts.

The ICA‐corrected data were then segmented into trials for the cue and feedback phases with a 2000 ms epoch (from 200 ms before cue/feedback onset to 1800 ms after onset) and for the donation phase with a 1200 ms epoch (from 200 ms before donation onset to 1000 ms after onset). The time window of 200 ms preceding the onset of the stimulus was used for baseline correction. Trials in which participants did not successfully receive monetary reward feedback were excluded from the analyses. Additionally, trials with voltage exceeding ±100 μV were visually inspected and removed. Only artifact‐free epochs were averaged across trials and participants for ERPs analysis. In total, 16.1%, 16.4%, and 11.5% of epochs were excluded from the cue, feedback, and donation phases, respectively.

Time‐frequency analysis was also conducted to calculate the ERSPs for each condition using EEGLAB. The ICA‐corrected data were segmented into trials from −600 ms pre‐stimulus to 1800 ms post‐stimulus during the cue and feedback phases, and from −600 ms pre‐donation to 1000 ms post‐donation during the donation phase. Artifact‐free EEG epochs underwent time‐frequency analysis using a short‐time Fast Fourier Transform with a fixed 400 ms Hanning‐tapered window, shifted in 10 ms steps. In the frequency domain, the spectrum was calculated from 1 to 20 Hz in increments of 0.5 Hz. The time interval from −400 to −200 ms before stimulus onset was used for baseline normalization. A total of 17.0%, 15.9%, and 9.4% of epochs were excluded from the cue, feedback, and donation phases, respectively.

### 2.7 EEG Analysis

The analyses of ERPs and ERSPs utilized specific time windows and electrode sites for each ERP of interest, guided by previous studies and topographical considerations. We focused on the P2 (cue‐P2) and CNV components in the cue phase, the RewP and fb‐P3 components in the feedback phase, and the P3 (donation‐P3) component in the donation phase.

The cue‐P2 component is characterized by a positive deflection that typically peaks around 200 ms after stimulus onset. It reflects greater attentional allocation to high‐value cues compared to non‐value or low‐value cues (Fröber et al. 2021; Nadig et al. 2019). We calculated the average EEG response for the cue‐P2 component from 200 to 320 ms post‐cue onset over the centro‐parietal electrodes (CP1, CPz, and CP2). The CNV component is a slow negative‐going waveform that occurs after cue onset and is observed over fronto‐central electrodes. It represents increased attentional and motor preparation for upcoming target stimuli following incentive cues compared to non‐incentive cues (Novak and Foti 2015; Wei and Ji 2021; Zhang et al. 2023). The EEG response for the CNV component was averaged between 800 and 1700 ms post‐cue onset over the fronto‐central electrodes (FC1, FCz, and FC2).

The RewP component is a positive deflection that peaks around 200–300 ms post‐feedback onset and serves as an index of individuals' sensitivity to gain (versus loss) outcomes (Meadows et al. 2016). We averaged the waveform in the time window of 200–280 ms post‐feedback onset over the fronto‐central electrodes (FC1, FCz, and FC2). The fb‐P3 component is a positive deflection that peaks between 300 and 600 ms after the feedback stimulus, reflecting outcome evaluation and feedback‐guided learning (Glazer et al. 2018; San Martín 2012). This component was defined from 300 to 500 ms post‐feedback onset over the parietal‐occipital electrodes (P1, Pz, and P2). Finally, the donation‐P3 component likely reflects the allocation of attentional and cognitive resources for calculating costs and benefits during prosocial decision‐making (Li et al. 2018). It was averaged within the time window of 500–600 ms post‐feedback onset over the centro‐parietal electrodes (CP1, CPz, and CP2). The amplitudes of these ERP components were extracted on a trial‐by‐trial basis for each condition and participant.

For the ERSP analyses in the cue phase, delta‐band power (cue‐delta, 1–4 Hz, 200–600 ms post‐cue onset) reflects attention allocated to motivationally salient stimuli (Gheza et al. 2018; Liu et al. 2020), while alpha‐band power (cue‐alpha, 8–12 Hz, 400–800 ms post‐cue onset) indicates preparation for upcoming stimuli (Jensen and Mazaheri 2010). These power measures were averaged over the centro‐parietal electrodes (CP1, CPz, and CP2). In the feedback phase, delta‐band power (fb‐delta, 1–4 Hz, 100–600 ms post‐feedback onset), which is assumed to relate to gain outcomes (Bachman et al. 2022; Cavanagh 2015), was averaged at the fronto‐central electrodes (FC1, FCz, and FC2). In the donation phase, both delta‐band power (donation‐delta, 1–4 Hz, 500–600 ms post‐donation offer onset) and theta‐band power (donation‐theta, 4–8 Hz, 180–300 ms post‐donation offer onset) were averaged at the centro‐parietal electrodes (CP1, CPz, and CP2). ERSP powers were extracted on a trial‐by‐trial basis for each condition and participant.

For the statistical analyses, LMMs were conducted on the ERPs and ERSPs for correct trials. Cue‐locked and feedback‐locked EEG models for each index (i.e., cue‐P2, CNV, cue‐delta, cue‐alpha, RewP, fb‐P3, and fb‐delta) were analyzed with effort type as a fixed factor. Similar models were applied to the donation‐locked EEG data (i.e., donation‐P3, donation‐delta, and donation‐theta), incorporating effort type, monetary cost, and their interactions as fixed factors. Significant main effects and interactions were further examined using post hoc tests with corrections for multiple comparisons where necessary. In all models, participant ID was included as a random intercept, with random slopes for the fixed effects to account for variance across individuals.

To further investigate how feedback‐locked EEG activity influenced subsequent prosocial behaviors, Spearman correlation analyses were conducted between differences in feedback‐locked EEG activity (effort minus no‐effort) and differences in donation acceptance rate (effort minus no‐effort). The RewP and fb‐P3 amplitudes for the correlation analyses were calculated as the mean values across all electrodes. A bootstrap procedure with 1000 samples was employed to determine the 95% credible interval of the correlation coefficient.

All LMM and GLMM statistical analyses were performed using R (Version 4.0.3) within R‐Studio (Version 2022.02.03). The lme4 package (http://CRAN.R‐project.org/package=lme4) was utilized for modeling, while the lmerTest package (http://CRAN.R‐project.org/package=lmerTest) was used for parameter testing. Post hoc tests with Bonferroni corrections were conducted using the emmeans package (https://CRAN.R‐project.org/package=emmeans). Correlation analyses were performed using IBM SPSS (Version 22.0). The significance level was set at *p* < 0.05.

## 3 Results

### 3.1 Behavioral Results

The probabilities of participants receiving reward feedback in the effort tasks in both experiments were 75.04% and 74.69%, respectively, ensuring a comparable number of trials to the no‐effort tasks and indicating that the adaptive manipulation was effective.

**FIGURE 2:**
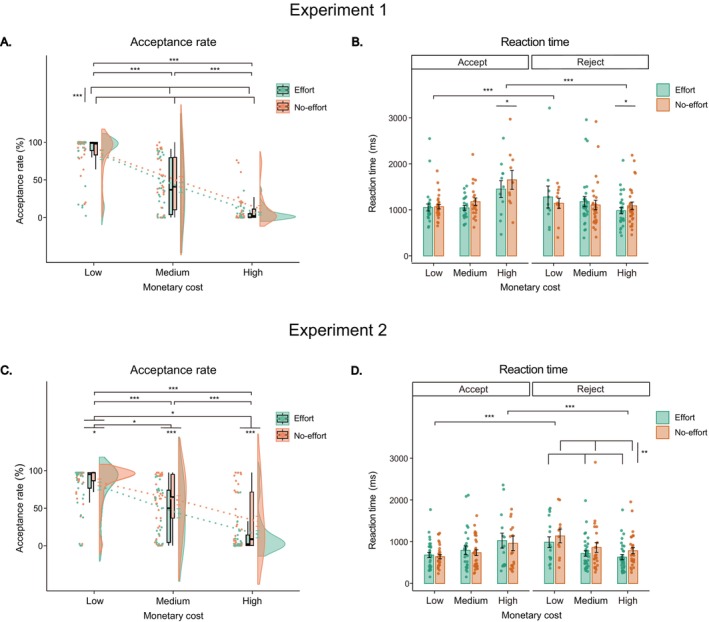
Behavioral results. (A, C) Acceptance rate results in Experiments 1 and 2, with green denoting effort conditions and orange denoting no‐effort conditions across three monetary cost levels (low, medium, high). Colored dots represent individual participant values, and boxplots indicate the median and 1st/3rd quartiles. Split‐half violin plots illustrate the probability density of data distribution, and green/orange dashed lines connect mean acceptance rates of effort/no‐effort conditions across low, medium, and high cost levels. (B, D) Mean response time results in Experiments 1 and 2 across experimental conditions, defined by effort type (effort, no‐effort), monetary cost (low, medium, high), and decision type (accept, reject). Error bars represent standard error of the mean (SE). Acceptance rates are percentages (%), and reaction times in milliseconds (ms). n.s. = non‐significant, *p < 0.05, **p < 0.01, ***p < 0.001 (after Bonferroni correction).

The GLMM results for acceptance rates in Experiment 1 (Figure 2A) revealed a significant main effect of effort type, *χ* ^2^(1) = 12.70, *p* < 0.001, *Φc* = 0.22. The acceptance rate in the effort condition was lower than that in the no‐effort condition (45% [IQR: 34%–61%] vs. 47% [IQR: 35%–65%], *b* = −0.70, *t* = −3.52, *p* < 0.001). Additionally, the main effect of monetary cost was significant, *χ* ^2^(2) = 175.12, *p* < 0.001, *Φc* = 0.80. The acceptance rate in the low‐cost condition was higher than that in the medium‐cost condition (98% [IQR: 89%–100%] vs. 42% [IQR: 7%–81%], *b* = 4.61, *t* = 10.56, *p* < 0.001) and was higher than that in the high‐cost conditions (2% [IQR: 0%–9%], *b* = 8.14, *t* = 13.11, *p* < 0.001). Furthermore, the acceptance rate in the medium‐cost condition was higher than that in the high‐cost condition (*b* = 3.53, *t* = 8.42, *p* < 0.001). The interaction between effort type and monetary cost was not significant, *χ* ^2^(2) = 1.16, *p* = 0.561.

The LMM results for RTs in Experiment 1 (Figure 2B) revealed a significant main effect of monetary cost, *F*(2, 8.24) = 22.78, *p* < 0.001, *η* ^2^_*p*_ = 0.85. RTs in the medium‐cost condition were faster than those in the low‐cost condition (1193 ± 322 vs. 1400 ± 512 ms, *b* = 206.80, *t* = 4.10, *p* < 0.001) and were faster than those in the high‐cost condition (1325 ± 337 ms, *b* = 131.90, *t* = 3.81, *p* < 0.001), whereas the RTs did not differ between the low‐cost and high‐cost conditions (*p* = 0.846). The interaction between effort type and monetary cost was also significant, *F*(2, 10.75) = 5.25, *p* = 0.026, *η* ^2^_*p*_ = 0.49. A simple effects test indicated that for high‐cost donation offers, RTs were faster in the effort condition than in the no‐effort condition (1163 ± 445 vs. 1488 ± 510 ms, *b* = 324.80, *t* = 2.52, *p* = 0.012). However, there was no significant difference in RTs between the effort and no‐effort conditions for the low‐cost donation offers (1434 ± 549 vs. 1367 ± 490 ms, *b* = 67.30, *t* = 1.85, *p* = 0.064) and medium‐cost donation offers (1167 ± 344 vs. 1219 ± 317 ms, *b* = 52.00, *t* = 1.76, *p* = 0.078). Additionally, there was a significant interaction between decision type and monetary cost, *F*(2, 7.92) = 39.98, *p* < 0.001, *η* ^2^_*p*_ = 0.91. Faster RTs were observed for acceptance compared to rejection in the low‐cost condition (1052 ± 284 vs. 1748 ± 945 ms, *b* = 695.40, *t* = 3.87, *p* < 0.001). Conversely, slower RTs were observed for acceptance compared to rejection in the high‐cost condition (1620 ± 505 vs. 1030 ± 367 ms, *b* = −589.90, *t* = −5.42, *p* < 0.001), while no significant difference was found between acceptance and rejection in the medium‐cost condition (1226 ± 402 vs. 1160 ± 495 ms, *b* = 66.20, *t* = 0.55, *p* = 0.580).

The GLMM results for acceptance rates in Experiment 2 (Figure 2C) revealed a similar pattern to that observed in Experiment 1. The main effect of effort type was significant, *χ* ^2^(1) = 21.94, *p* < 0.001, *Φc* = 0.28, with a lower acceptance rate in the effort condition than that in the no‐effort condition (52% [IQR: 32%–59%] vs. 57% [IQR: 46%–79%], *b* = −1.94, *t* = −4.48, *p* < 0.001). Additionally, the main effect of monetary cost was significant, *χ* ^2^(2) = 41.67, *p* < 0.001, *Φc* = 0.39. The acceptance rate in the low‐cost condition was higher than that in the medium‐cost condition (98% [IQR: 73%–100%] vs. 62% [IQR: 24%–77%], *b* = 3.88, *t* = 6.08, *p* < 0.001) and was higher than that in the high‐cost condition (9% [IQR: 1%–41%], *b* = 6.93, *t* = 6.46, *p* < 0.001). Furthermore, the acceptance rate in the medium‐cost condition was higher than that in the high‐cost condition (*b* = 3.05, *t* = 5.92, *p* < 0.001). There was also a significant interaction between effort type and monetary cost, *χ* ^2^(2) = 19.42, *p* < 0.001, *Φc* = 0.27. The differences in acceptance rates between the effort and no‐effort conditions were larger in the high‐cost (*p* = 0.012) and medium‐cost conditions (*p* = 0.017) than in the low‐cost condition, while the acceptance rate difference between high‐cost and medium‐cost conditions was not significant (*p* = 0.863).

The LMM results for RTs in Experiment 2 (Figure 2D) also revealed a significant main effect of monetary cost, *F*(2, 13.31) = 7.42, *p* = 0.007, *η* ^2^_*p*_ = 0.53. RTs in the medium‐cost condition were faster than those in the low‐cost condition (764 ± 371 vs. 838 ± 409 ms, *b* = 74.30, *t* = 3.48, *p* < 0.001) and were faster than those in the high‐cost condition (826 ± 445 ms, *b* = 62.30, *t* = 2.56, *p* = 0.031), while the RTs did not differ between the low‐cost and high‐cost conditions (*p* = 0.981). There was a significant interaction between effort type and decision type, *F*(1, 13.54) = 7.48, *p* = 0.017, *η* ^2^_*p*_ = 0.36. A simple effects test indicated that participants took less time to reject donation offers in the effort condition compared to the no‐effort condition (777 ± 395 vs. 866 ± 472 ms, *b* = 89.30, *t* = 2.82, *p* = 0.005). However, there was no significant difference in RTs for accepting donation offers between the effort and no‐effort conditions (805 ± 418 vs. 789 ± 427 ms, *b* = 16.10, *t* = 0.54, *p* = 0.592). Additionally, there was a significant interaction between decision type and monetary cost, *F*(2, 14.89) = 10.97, *p* < 0.001, *η* ^2^_*p*_ = 0.60. Faster RTs were observed for acceptance compared to rejection in the low‐cost condition (644 ± 290 vs. 1032 ± 604 ms, *b* = 388.51, *t* = 4.63, *p* < 0.001). Conversely, slower RTs were observed for acceptance compared to rejection in the high‐cost condition (982 ± 642 vs. 670 ± 343 ms, *b* = −311.95, *t* = −3.40, *p* < 0.001), while no significant difference was observed between acceptance and rejection in the medium‐cost condition (766 ± 399 vs. 762 ± 343 ms, *b* = 3.93, *t* = 0.07, *p* = 0.946).

### 3.2 HDDM Results in Experiment 1

The DIC values indicated the winning model is Model 3, which is the most complex model allowing both parameters of interest (*v* and *a*) to vary by effort type and monetary cost (Table 1).

**TABLE 1:** Overview of the models estimated and their DIC values.

Model	Formula	DIC
1	*v*~effort type, monetary cost	8056.07
2	*a*~effort type, monetary cost	13179.68
3	*v*, *a*~effort type, monetary cost	7881.47

**FIGURE 3:**
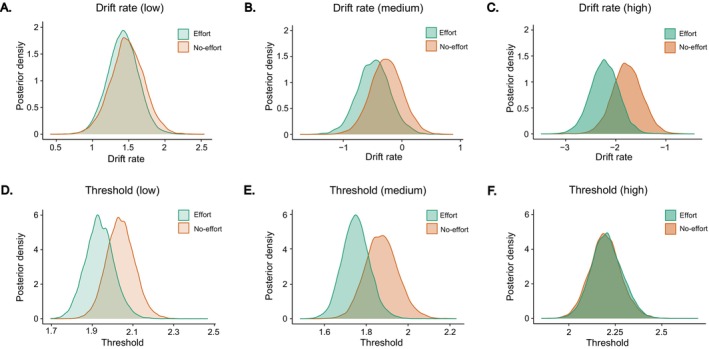
Hierarchical drift diffusion model results. (A–C) Posterior probability distribution of drift rate (*v*) comparing effort and no‐effort conditions across low‐, medium‐, and high‐cost donation offers, respectively. (D–F) Posterior probability distribution of threshold (*a*) comparing effort and no‐effort conditions across low‐, medium‐, and high‐cost donation offers, respectively.

The HDDM analysis revealed that the drift rate (*v*) was not reliably different between the effort and no‐effort conditions for the low‐cost donation offers (*b* = 0.06, 95% CI [−0.13, 0.25], *P*_posterier_ (effort > no‐effort) = 0.701, Figure 3A) and medium‐cost donation offers (*b* = −0.22, 95% CI [−0.64, 0.20], *P*_posterier_ (effort < no‐effort) = 0.801, Figure 3B). Importantly, as shown in Figure 3C, participants were more inclined to reject high‐cost donation offers under the effort condition compared to the no‐effort condition, and they overall responded faster, as it is indicated by the more negative drift rate in the effort condition (*b* = −1.74, 95% CI [−0.93, −0.02], *P*_posterier_ (effort < no‐effort) = 0.992).

Moreover, the analyses of decision threshold (*a*) revealed that participants in the effort condition had a lower decision threshold compared to the no‐effort condition for low‐cost donation offers (*b* = −0.10, 95% CI [−0.18, −0.02], *P*_posterier_ (effort < no‐effort) = 0.984, Figure 3D) and medium‐cost donation offers (*b* = −0.12, 95% CI [−0.22, −0.03], *P*_posterier_ (effort < no‐effort) = 0.986, Figure 3E). However, there was no evidence for a difference between the effort and no‐effort conditions for high‐cost donation offers (*b* = −0.01, 95% CI [−0.12, 0.14], *P*_posterier_ (effort < no‐effort) = 0.466, Figure 3F).

### 3.3 EEG Results in Experiment 2

#### 3.3.1 Cue Phase

**FIGURE 4:**
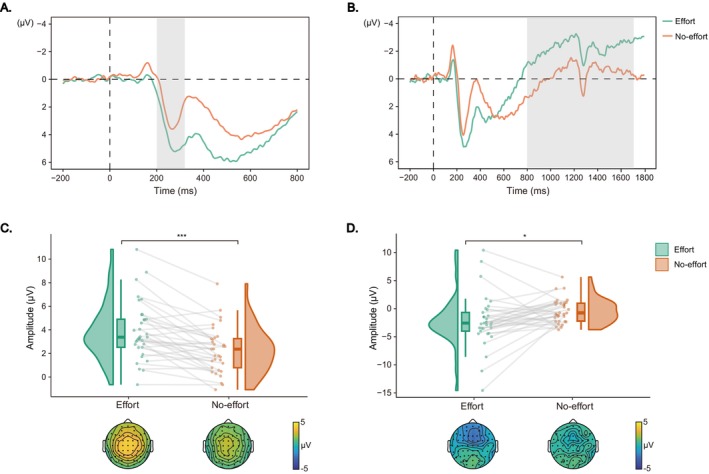
Cue‐locked ERPs results. (A, B) Grand‐averaged ERP waveforms for the cue‐P2 and CNV components, with gray shaded areas denoting the time windows of interest. (C, D) Raincloud plots displaying mean amplitudes and topographic maps for the cue‐P2 and CNV components, respectively. Each dot indicates per‐participant average amplitude for each condition, and gray solid lines connect data points of the same participant across conditions. Boxplots display group‐level statistics (median, 1st/3rd quartiles), and split‐half violin plots illustrate the probability density of data distribution. *p < 0.05, ***p < 0.001 (after Bonferroni correction).

For the ERPs in the cue phase, significant main effects of effort type were observed for both the cue‐P2 (Figure 4A,C), *F*(1, 31.63) = 29.07, *p* < 0.001, *η* ^2^_*p*_ = 0.48, and the CNV components (Figure 4B,D), *F*(1, 31.99) = 5.95, *p* = 0.020, *η* ^2^_*p*_ = 0.16. Specifically, the effort condition exhibited more positive‐going cue‐P2 waveforms (3.88 ± 2.44 vs. 2.28 ± 1.90 μV) and more negative‐going CNV waveforms (−2.21 ± 4.62 vs. −0.37 ± 2.13 μV) compared to the no‐effort condition.

**FIGURE 5:**
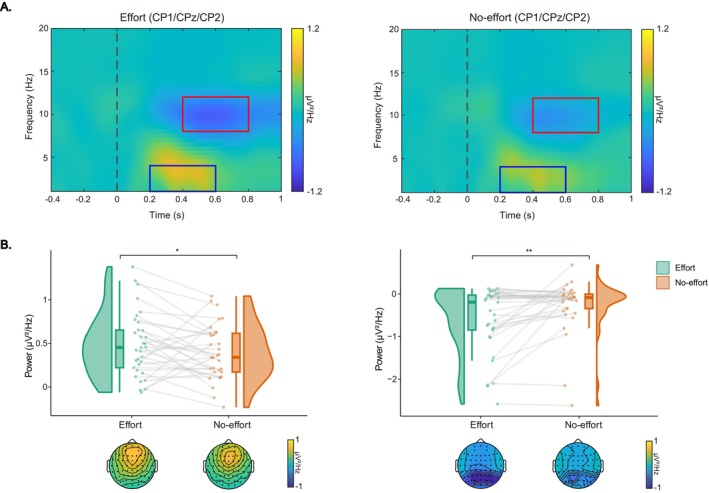
Cue‐locked ERSPs results. (A) Grand‐averaged ERSP power for cue‐delta (blue boxes) and cue‐alpha (red boxes) for the effort and no‐effort conditions. (B) Raincloud plots displaying mean power and topographic maps for cue‐delta (left) and cue‐alpha (right) bands, respectively. Each dot indicates per‐participant average amplitude for each condition, and gray solid lines connect data points of the same participant across conditions. Boxplots display group‐level statistics (median, 1st/3rd quartiles), and split‐half violin plots illustrate the probability density of data distribution. *p < 0.05, **p < 0.01 (after Bonferroni correction).

For the ERSPs in the cue phase, significant main effects of effort type were observed for both the cue‐delta band power, *F*(1, 30.05) =4.29, *p* = 0.047, *η* ^2^_*p*_ = 0.10, and cue‐alpha band power, *F*(1, 33.39) = 9.05, *p* = 0.005, *η* ^2^_*p*_ = 0.21. The cue‐delta band power (indicated by blue boxes in Figure 5A) was higher in the effort condition compared to the no‐effort condition (0.49 ± 0.38 vs. 0.38 ± 0.32 μV^2^/Hz). In contrast, the cue‐alpha band power (indicated by red boxes in Figure 5A) was lower in the effort condition than in the no‐effort condition (−0.56 ± 0.74 vs. −0.25 ± 0.59 μV^2^/Hz, Figure 5B).

#### 3.3.2 Feedback Phase

**FIGURE 6:**
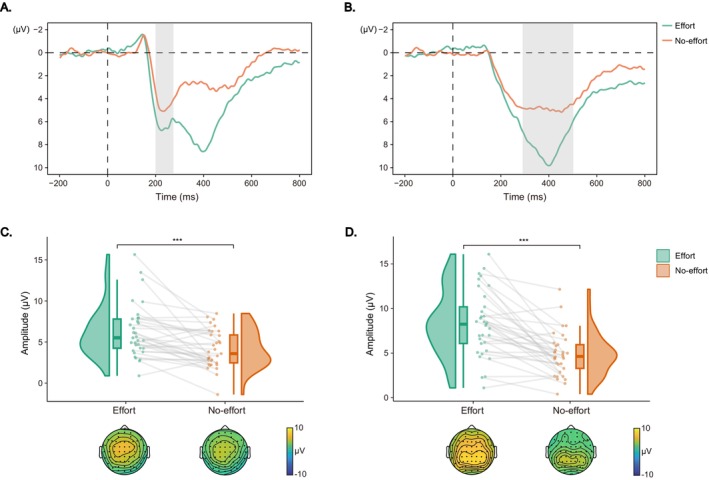
Feedback‐locked ERPs results. (A, B) Grand‐averaged ERP waveforms for the RewP and fb‐P3 components. The gray shaded areas indicate the time windows of interest. (C, D) Raincloud plots displaying mean amplitudes and topographic maps for the RewP and fb‐P3 components, respectively. Each dot indicates per‐participant average amplitude for each condition, and gray solid lines connect data points of the same participant across conditions. Boxplots display group‐level statistics (median, 1st/3rd quartiles), and split‐half violin plots illustrate the probability density of data distribution. ***p < 0.001 (after Bonferroni correction).

For the RewP component during the feedback phase (Figure 6A,C), there was a significant main effect of effort type, *F*(1, 32.07) = 13.06, *p* < 0.001, *η* ^2^_*p*_ = 0.29, with larger amplitudes in the effort condition compared to the no‐effort condition (6.31 ± 3.13 vs. 4.60 ± 2.40 μV). Similarly, the analysis on the fb‐P3 component (Figure 6B,D) revealed a significant main effect of effort type, *F*(1, 32.68) = 34.51, *p* < 0.001, *η* ^2^_*p*_ = 0.51, with larger amplitudes in the effort condition than in the no‐effort condition (8.32 ± 3.67 vs. 4.90 ± 2.44 μV).

**FIGURE 7:**
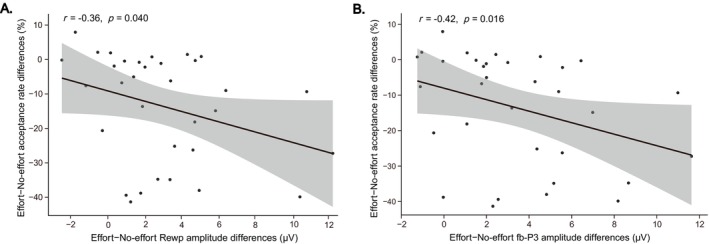
Correlation analysis results. (A) Scatter plot depicting the relationship between RewP amplitude differences (effort minus no‐effort) and acceptance rate differences (effort minus no‐effort); (B) Scatter plot illustrating the relationship between fb‐P3 amplitude differences (effort minus no‐effort) and acceptance rate differences (effort minus no‐effort). Shaded areas indicate 95% confidence intervals for the regression lines.

Correlation analysis revealed a significant correlation coefficient between the RewP amplitude differences and the differences in donation acceptance rates between the effort and no‐effort conditions (*r* = −0.36, *p* = 0.040, 95% CI [−0.67, −0.02]; Figure 7A). Additionally, a significant correlation was observed between the fb‐P3 amplitude differences and the differences in donation acceptance rates between the effort and no‐effort conditions (*r* = −0.42, *p* = 0.016, 95% CI [−0.66, −0.09]; Figure 7B).

**FIGURE 8:**
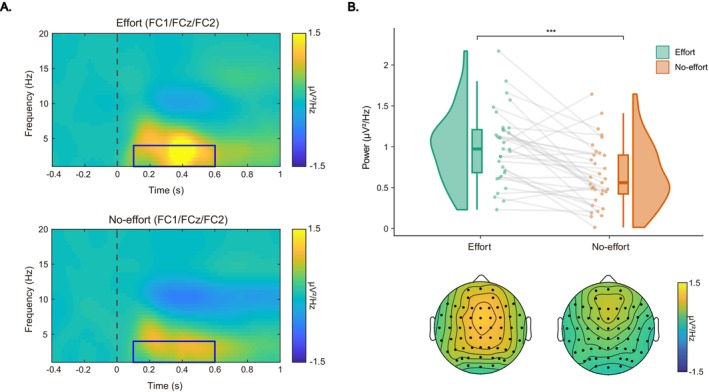
Feedback‐locked ERSPs results. (A) Grand‐averaged ERSP power of the fb‐delta band (blue boxes) for effort and no‐effort conditions. (B) Raincloud plots displaying mean fb‐delta power and topographic maps. Each dot indicates the per‐participant average amplitude for each condition, and gray solid lines connect data points of the same participant across conditions. Boxplots display group‐level statistics (median, 1st/3rd quartiles), and split‐half violin plots illustrate the probability density of the data distribution. ***p < 0.001 (after Bonferroni correction).

For the fb‐delta band power during the feedback phase (as indicated by blue boxes in Figure 8A), a significant main effect of effort type was observed, *F*(1, 33.39) = 21.74, *p* < 0.001, *η* ^2^_*p*_ = 0.39, with higher power in the effort condition than that in the no‐effort condition (0.99 ± 0.44 vs. 0.66 ± 0.38 μV^2^/Hz, Figure 8B).

#### 3.3.3 Donation Phase

**FIGURE 9:**
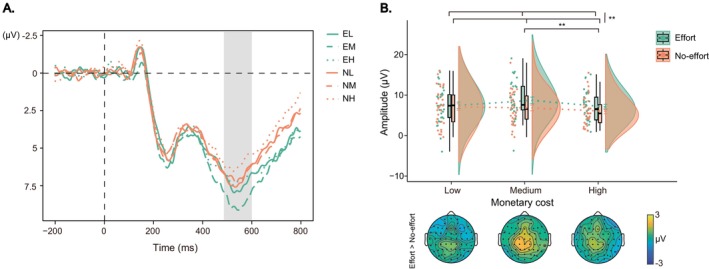
Donation‐locked ERP results. (A) Grand‐averaged ERP waveforms for the P3 components, with gray shaded areas indicating the specified time window. (B) Raincloud plots displaying mean amplitudes and topographic maps for the P3 component. Colored dots indicate individual participant values, and boxplots denote the median and 1st/3rd quartiles. Split‐half violin plots display the probability density of data distribution, and green/orange dashed lines connect the mean values of effort/no‐effort conditions across low, medium, and high cost levels (EH, effort high‐cost; EL = effort low‐cost; EM, effort medium‐cost; NH, no‐effort high‐cost; NL, no‐effort low‐cost; NM, no‐effort medium‐cost). **p < 0.01 (after Bonferroni correction).

Analysis on the donation‐P3 component during the donation phase (Figure 9A,B) revealed a significant main effect of effort type, *F*(1, 33.32) = 6.87, *p* = 0.013, *η* ^2^_*p*_ = 0.17, with larger amplitudes in the effort condition compared to the no‐effort condition (7.93 ± 3.90 vs. 6.77 ± 3.64 μV). The main effect of monetary cost was also significant, *F*(2, 30.73) = 5.12, *p* = 0.012, *η* ^2^_*p*_ = 0.25, with larger amplitudes observed in the medium‐cost condition compared to the high‐cost condition (7.92 ± 3.81 vs. 6.66 ± 3.52 μV, *b* = 1.25, *t* = 3.20, *p* = 0.004). However, no significant differences were observed between the low‐cost condition and the other conditions, *ps* > 0.05. The interaction between effort type and monetary cost was not significant, *p* > 0.05.

**FIGURE 10:**
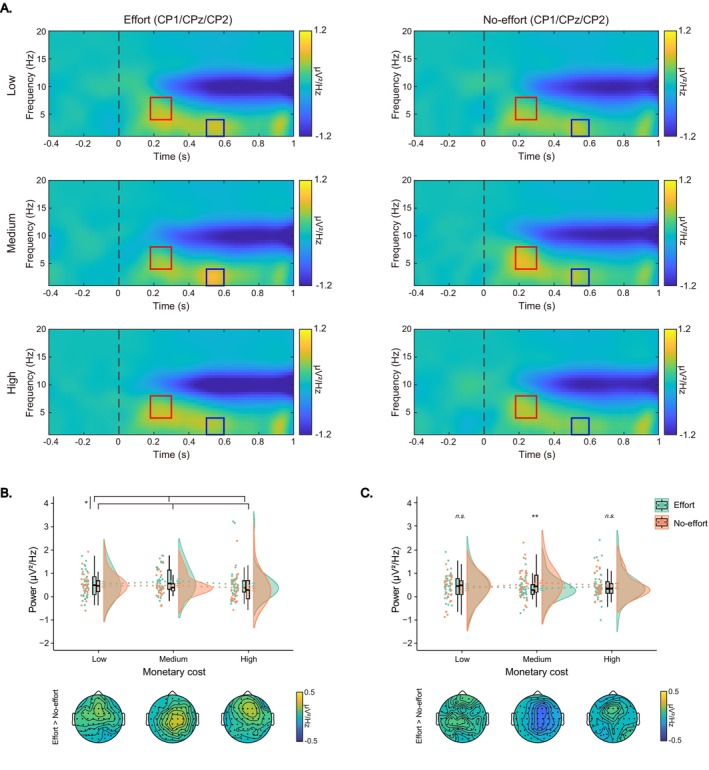
Donation‐locked ERSPs results. (A) Grand‐averaged ERSP power for donation‐delta (blue boxes) and donation‐theta (red boxes) across each condition. (B, C) Raincloud plots displaying mean power and topographic maps for donation‐delta and donation‐theta, respectively. Colored dots indicate individual participant values, and boxplots denote the median and 1st/3rd quartiles. Split‐half violin plots display probability density of the data distribution, and green/orange dashed lines connect the mean values of effort/no‐effort conditions across low, medium, and high cost levels. n.s. non‐significant, *p < 0.05, **p < 0.01 (after Bonferroni correction).

For the delta band power during the donation phase (as indicated by blue boxes in Figure 10A), a significant main effect of effort type was observed, *F*(1, 54.29) = 4.81, *p* = 0.032, *η* ^2^_*p*_ = 0.08, with higher power in the effort condition compared to the no‐effort condition (0.53 ± 0.42 vs. 0.40 ± 0.41 μV^2^/Hz, Figure 10B). In contrast, for the theta band power during the donation phase (red boxes in Figure 10A), neither the main effect of effort type nor the main effect of monetary cost was significant (*ps* > 0.05). However, the interaction between effort type and monetary cost was significant, *F*(2, 80.25) = 3.94, *p* = 0.019, *η* ^2^_*p*_ = 0.09. In the medium‐cost condition, power in the effort condition was lower than in the no‐effort condition (0.29 ± 0.54 vs. 0.55 ± 0.51 μV^2^/Hz, *b* = −0.26, *t* = −2.71, *p* = 0.007). Conversely, the power between the effort and no‐effort conditions was comparable in the low‐cost (0.41 ± 0.51 vs. 0.30 ± 0.48 μV^2^/Hz, *b* = 0.11, *t* = 1.15, *p* = 0.251) and the high‐cost (0.41 ± 0.58 vs. 0.42 ± 0.55 μV^2^/Hz, *b* = −0.01, *t* = −0.02, *p* = 0.982) conditions (Figure 10C).

## 4 Discussion

In the current study, we manipulated the sources of monetary gains (effort vs. no‐effort) and the levels of monetary costs (low, medium, high) associated with prosocial donation decisions to elucidate the cognitive and neural dynamics underlying the influence of prior effort expenditure on subsequent prosocial decision‐making. Our findings demonstrated that effort expenditure and monetary costs jointly shape prosocial decision‐making at multiple levels. Behaviorally, both experiments revealed that participants were less willing to donate money earned through effort compared to money obtained effortlessly, particularly under higher monetary cost conditions. They also responded more quickly to reject high‐cost donation offers than to accept them, indicating a stronger prosocial apathy in these situations (Carlsson et al. 2013; Hernandez Lallement et al. 2014; Li et al. 2019). Computationally, HDDM results revealed faster drift rates (*v*) and lower decision thresholds (*a*) in the effort conditions compared to the no‐effort conditions, suggesting a more efficient accumulation of self‐interest evidence and a simplified decision‐making process shaped by cost‐dependent motivational conflict. Neurally, the enhanced RewP, fb‐P3 amplitudes, and fb‐delta power following monetary feedback in the effort condition suggest that heightened reward sensitivity associated with money earned through effort may contribute to prosocial apathy. Furthermore, the reduced theta power in the effort condition compared to the no‐effort condition during donation evaluation, particularly for medium‐cost offers, corresponded with lower decision thresholds in behavioral responses. This suggests that effort mitigated response conflicts and facilitated proself decisions. Together, these results provide converging behavioral, computational, and neural evidence that effort expenditure amplifies reward evaluation and simplifies decision‐making, reinforcing self‐serving choices in prosocial contexts.

Effort is certainly a cost to consider when evaluating prosocial behavior. However, despite decades of research employing various methods to assess prosocial tendencies, such as self‐reports and economic games, the experimental manipulation of effort has largely been overlooked until recently (Contreras‐Huerta et al. 2020). As mentioned in the Introduction, recent studies have created scenarios in which participants choose to exert effort to benefit others, revealing a general reluctance to exert effort for others compared to oneself (Contreras‐Huerta et al. 2022; Depow et al. 2022; Forbes et al. 2024; Lockwood, Ang, et al. 2017; Lockwood et al. 2022; Talbot et al. 2025). While these studies primarily focused on effort expenditure during prosocial behavior, the current study manipulates whether the source of monetary gain is effortful or not prior to engaging in prosocial behavior. Although there is evidence that sensitivity to monetary rewards can be separated from sensitivity to effort (Le Heron et al. 2018; Varazzani et al. 2015), both lines of research point to the phenomenon of prosocial apathy when effort is considered a cost in prosocial behavior. Our findings extend existing behavioral evidence (Hernandez Lallement et al. 2014; Li et al. 2021) by demonstrating that brain responses to monetary feedback following effort expenditure are predictive of individuals' prosocial behavior.

Consistent with previous research (Bogdanov et al. 2022; Harmon‐Jones, Clarke, et al. 2020; Harmon‐Jones, Willoughby, et al. 2020; Ma et al. 2014; Yi et al. 2020; but see Bowyer et al. 2021; Gheza et al. 2018; Wu and Zheng 2023), we found that exerting effort increased the magnitude of the RewP and fb‐P3 amplitudes in response to monetary gains, suggesting enhanced reward sensitivity and affective significance following physical effort expenditure. Alongside the results of enhanced fb‐delta power associated with performance and reward evaluation (Cavanagh 2015; Foti et al. 2015; Leicht et al. 2013; Pornpattananangkul and Nusslock 2016), our findings support the notion that the subjective value of rewards is elevated when they are obtained through effort (Norton et al. 2012). This increased value may reflect a drive to resolve cognitive dissonance by justifying effort invested (Harmon‐Jones, Clarke, et al. 2020; Harmon‐Jones, Willoughby, et al. 2020; Harmon‐Jones et al. 2024), as the exertion of effort can produce an aversive state that makes subsequent rewards appear more valuable in comparison (Inzlicht et al. 2018).

The reduced willingness to donate money earned through effort, as opposed to windfall gains, may be explained by two factors. First, effort‐derived earnings may carry a stronger sense of personal justification compared to windfall money, leading to enhanced affective significance for the earned amount and a greater inclination to retain it for oneself. Our analysis revealed that the differences in donation acceptance rates between the effort and no‐effort conditions were negatively correlated with differences in RewP and fb‐P3 amplitudes. These findings suggest that increased sensitivity and affective significance to monetary rewards obtained through effort may contribute to greater prosocial apathy. This is consistent with recent research indicating that individuals who accumulated larger sums in the Effort‐Expenditure for Rewards Task, reflecting higher sensitivity to monetary rewards, tended to donate a smaller share of their total earnings (Mao et al. 2024).

Another explanation is that participants may experience a state of diminished capacity due to fatigue resulting from effort expenditure, making it more challenging for them to engage in additional effortful behavior. Previous studies have suggested that an individual's capacity plays a significant role in their engagement in prosocial behavior, such as empathy (Contreras‐Huerta et al. 2020; Lockwood, Ang, et al. 2017; Lockwood, Hamonet, et al. 2017). Additionally, prosocial behavior incurs both cognitive and emotional costs (Cameron et al. 2019; Contreras‐Huerta et al. 2020). People often feel exhausted by social situations that require empathy (Cameron et al. 2016; Guyett 2007; Hansen et al. 2018; Klimecki and Singer 2012), much like the fatigue reported after engaging in physically or cognitively demanding activities (Müller and Apps 2019). As a result, after experiencing fatigue from button‐pressing, participants may find it more difficult to donate money when doing so requires empathizing with people in need, compared to conditions with no effort expended beforehand.

Furthermore, not only is prosocial behavior costly, but carefully weighing the self‐interest versus the donation share during the decision‐making process can also be effortful, particularly after continuous button pressing. Therefore, a quick rejection decision, especially in the high‐cost conditions after effort expenditure, as evidenced by faster drift rate and RTs, may help alleviate this “suffering.” Moreover, the lower decision thresholds in the effort condition compared to the no‐effort condition further indicate a cognitive simplification in the decision‐making process, as participants reduced the amount of evidence needed to reach a decision. This supports the idea that the costs of mental effort discourage the inefficient allocation of cognitive control for detailed decision‐making that may itself be computationally intensive (Kool and Botvinick 2013). The increased P3 amplitudes and enhanced delta power in the effort condition compared to the no‐effort condition during the donation evaluation phase support the notion that effortful processes lead to increased attentional control in the evaluating of costs and benefits. This is based on the assumption that these neural activities are associated with the allocation of greater attentional and cognitive resources for assessing costs and benefits during prosocial decision‐making (Bachman and Bernat 2018; Güntekin and Başar 2016; Li et al. 2018; Li et al. 2021).

The effects of this cognitive simplification on explicit prosocial behavior varied across contexts, influenced by the degree of monetary cost and motivational conflict. Past research on prosocial decisions has indicated that participants' choices are driven by both proself and prosocial motives (Falco et al. 2023; Numano et al. 2024). In our study, these two motives may be dynamically modulated by effort type and monetary cost levels. In the low‐cost condition, prosocial motives that favored the acceptance of low‐cost offers were predominant, resulting in weak motivational conflict. As a consequence, participants consistently exhibited generosity, which was manifested by high overall acceptance rates. Notably, reaction times did not differ between the two effort conditions. Although effort appeared to streamline the decision‐making process (as evidenced by lower decision thresholds), it did not translate into faster behavioral responses. This likely underscores the powerful influence of prosocial motives in the low‐cost condition. In contrast, the medium‐cost condition may activate comparable proself and prosocial motives, leading to stronger motivational conflict. As mentioned earlier, effort expenditure can prioritize self‐protective heuristic behaviors and justify personal earnings, thereby amplifying the salience of self‐interest and diminishing thoughtful consideration of others (Harmon‐Jones, Clarke, et al. 2020; Kurzban et al. 2013). Meanwhile, the reduced theta power observed in the effort condition relative to the no‐effort condition, particularly under medium‐cost offers, provides additional evidence for this cognitive simplification process. The theta oscillations are widely regarded as neural markers of conflict monitoring and cognitive control demands (Cavanagh et al. 2012; Sauseng et al. 2010). The lower theta power suggests that effort expenditure diminishes the need for conflict monitoring when evaluating medium‐cost offers, consistent with the lower decision thresholds. Additionally, the high‐cost condition was associated with even greater motivational conflict (see also Li et al. 2021), as the increased personal monetary cost amplified proself motives. In this context, the complex trade‐offs inherent in decision‐making, regardless of effort exertion, likely resulted in no significant changes in decision thresholds. Coupled with the higher drift rate in the effort condition relative to the no‐effort condition when rejecting high‐cost offers, these findings suggest that effort expenditure in high‐cost scenarios heightened the salience of self‐interest, resulting in expedited rejection decisions.

To fully characterize the neural dynamics of effort processing, it is essential to investigate the antecedent preparatory phase in which the brain orchestrates attentional and motivational resources prior to effort exertion and reward delivery. Importantly, the electrophysiological results during the effort anticipation phase in Experiment 2 support the notion that participants exhibited greater attentional control for effort trials relative to no‐effort trials. The enhanced cue‐P2 (Schevernels et al. 2014; Zhang et al. 2023) and cue‐delta power (Knyazev 2007; Stefanics et al. 2010) at an early stage suggest enhanced attention allocation, and the enhanced cue‐CNV (Frömer et al. 2021; Jia et al. 2021; Novak and Foti 2015; Schevernels et al. 2014; Wei and Ji 2021; Wu et al. 2019) and cue‐alpha power (Jensen and Mazaheri 2010; van Driel et al. 2012) at a later stage suggested stronger motivational preparation for the upcoming task. These results are consistent with the majority of previous studies reporting these components.

However, some comparisons with previous studies should be highlighted. For example, the cue‐P2 has been documented to correlate with rapid recognition and classification of cues, reflecting the allocation of attentional resources to stimuli (Schevernels et al. 2014; Zhang et al. 2023). Falkenstein and colleagues reported that a larger P2 was elicited for effort trials than for standard trials (Falkenstein et al. 2003). However, their study introduced a confounding factor: the probability of effort trials was lower than that of standard trials, potentially increasing the salience of effort trials and leading to greater attentional allocation. Expanding their work, we manipulated the probabilities for effort trials a bit higher than that for no‐effort trials in the current study and still observed the P2 effect between the effort and no‐effort conditions, indicating that effort cues indeed captured more attentional resources relative to no‐effort cues.

Moreover, although most studies reported more negative CNV in conditions with higher rewards or those requiring enhanced task preparation (Frömer et al. 2021; Jia et al. 2021; Novak and Foti 2015; Schevernels et al. 2014; Wei and Ji 2021; Wu et al. 2019), which is consistent with our CNV findings. These results are in contrast with those of a recent study by Zhang and colleagues, which combined effort and reward information in a modified MID task (Zhang and Zheng 2022). Zhang and colleagues reported that the CNV was enhanced for the low‐effort condition compared to the high‐effort condition, which stands in contrast to our CNV results. These conflicting findings may be attributed to methodological differences between the two studies. In the Zhang et al. study, the success rate was manipulated to hover around 50% across conditions, whereas in the current study, the success rate for effort trials (75% positive feedback) was lower compared to no‐effort trials (100% positive feedback). As a result, participants in the current study were assured monetary rewards with minimal effort in the no‐effort condition, while in the effort condition they needed to activate stronger motivational responses and engage in more effective task preparation to achieve a higher accuracy rate in order to receive monetary rewards. This difference in task demands may account for the observed discrepancies in CNV results between the two studies. Notably, despite this methodological variation, our findings are consistent with the broader principle that effort cues mobilize attentional and motivational resources. The anticipatory neural responses we observed indicate that effort cues trigger proactive adjustments in attention and motivation. This in turn serves as the basis for heightened reward sensitivity and simplified decision‐making processes observed in subsequent stages.

In conclusion, our findings extend and substantiate the computational framework of evidence accumulation for voluntary prosocial decision‐making influenced by effort expenditure and monetary costs. Our results provide evidence that the exertion of physical effort increases electrophysiological correlates of neural activity during both effort cuing and reward feedback processing stages. Notably, correlational analyses revealed that participants exhibited heightened reward sensitivity to monetary gains obtained through effort, which corresponded to less generous prosocial behavior. Collectively, these results illuminate the behavioral and neural mechanisms of effort expenditure and monetary costs on prosocial decision‐making.

## Data Availability

The data that support the findings of this study are available from the corresponding author upon reasonable request.
